# Modeling the *Mycobacterium tuberculosis* Granuloma – the Critical Battlefield in Host Immunity and Disease

**DOI:** 10.3389/fimmu.2013.00098

**Published:** 2013-04-22

**Authors:** Evelyn Guirado, Larry S. Schlesinger

**Affiliations:** ^1^Department of Microbial Infection and Immunity, Center for Microbial Interface Biology, The Ohio State UniversityColumbus, OH, USA

**Keywords:** *Mycobacterium tuberculosis*, model, granuloma, tuberculosis, pathogenesis

## Abstract

Granulomas are the hallmark of *Mycobacterium tuberculosis* (*M.tb*) infection and thus sit at the center of tuberculosis (TB) immunopathogenesis. TB can result from either early progression of a primary granuloma during the infection process or reactivation of an established granuloma in a latently infected person. Granulomas are compact, organized aggregates of immune cells consisting of blood-derived infected and uninfected macrophages, foamy macrophages, epithelioid cells (uniquely differentiated macrophages), and multinucleated giant cells (Langerhans cells) surrounded by a ring of lymphocytes. The granuloma’s main function is to localize and contain *M.tb* while concentrating the immune response to a limited area. However, complete eradication does not occur since *M.tb* has its own strategies to persist within the granuloma and to reactivate and escape under certain conditions. Thus *M.tb*-containing granulomas represent a unique battlefield for dictating both the host immune and bacterial response. The architecture, composition, function, and maintenance of granulomas are key aspects to study since they are expected to have a profound influence on *M.tb* physiology in this niche. Granulomas are not only present in mycobacterial infections; they can be found in many other infectious and non-infectious diseases and play a crucial role in immunity and disease. Here we review the models currently available to study the granulomatous response to *M.tb*.

## Introduction

An estimated one-third of the world’s population carries an asymptomatic infection with *Mycobacterium tuberculosis* (*M.tb*), which results in eight million new cases of tuberculosis (TB) and two million deaths every year (WHO, 2011). Granulomas are the hallmark of *M.tb* infection and thus sit at the center of TB immunopathogenesis. TB can result from either early progression of a primary granuloma during the infection process (rare) or reactivation of an established granuloma in a latently infected person (10% lifetime risk in an otherwise healthy individual).

**Figure 1:**
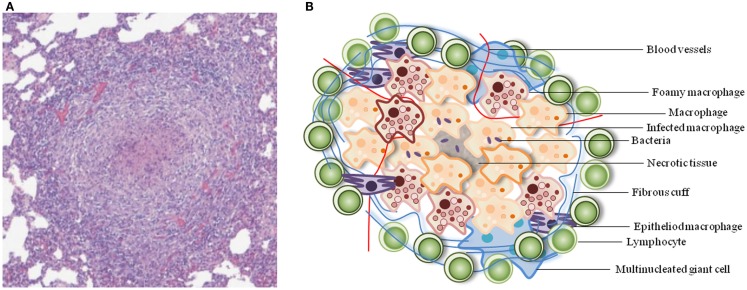
**Typical architecture of a TB granuloma**. **(A)** Representative granuloma with central necrosis from minipig lung tissue. Histological samples were formalin-fixed, cut, and stained with hematoxylin-eosin. Adapted from Gil et al. (2010). **(B)** Schematic of the cellular constituents of a TB granuloma.

Granulomas are well-organized, dynamic structures with immune cells at various stages of differentiation (Figure 1). The cellular composition of TB granulomatous lesions includes blood-derived infected and uninfected macrophages, foamy macrophages, epithelioid cells (uniquely differentiated macrophages), and multinucleated giant cells (Langerhans cells), B and T lymphocytes, and fibroblasts (Russell, 2007; Ramakrishnan, 2012).

In human pulmonary TB, the granuloma formation process starts shortly after infection. When inhaled, *M.tb* is ingested by and transported across the alveolar epithelium by AMs into the lung tissue and adjacent lymph nodes, and then dissemination ensues through the lymphatics and blood stream. This process initiates a cascade of events involving the production of pro and anti-inflammatory cytokines and chemokines. The immune response generated stimulates the activation of phagocyte anti-microbial activities and leads to the recruitment of additional mononuclear leukocytes into the site of infection. This accumulation of cells around the foci of infected cells leads to the formation of a macrophage-rich cell mass known as a granuloma.

*Mycobacterium tuberculosis* can persist for decades within the granuloma structures and, due to some intervening medical (e.g., HIV infection, diabetes, cancer, malnutrition, aging, etc.) and/or genetic factors, bacteria can reactivate. A balance of pro-inflammatory and anti-inflammatory immune responses is essential for controlling bacterial proliferation within granulomas and the resolution of these granuloma lesions over time. A dysregulation in the immune response will lead to granuloma progression and disease. An accumulation of caseum in the center of the granuloma promotes an increase in necrotic tissue and the collapse of the granuloma center which releases virulent bacilli to other parts of the tissue where more lesions will be formed. In the lungs, breakdown of the granuloma into the airways can lead to transmission of bacteria to other individuals.

In general, the production of chemokines is essential for the recruitment of inflammatory cells to the site of infection (Algood et al., 2003). Particularly, the chemokines binding to the CCR2 receptor (CCL2/MCP-1, CCL12, and CCL13) play an essential role for the early recruitment of macrophages. During the early stages of granuloma formation, TNF produced by infected macrophages and T cells plays a crucial role in maintaining the granuloma structure by keeping sustained levels of chemokines and cellular recruitment and retention (Roach et al., 2002; Chakravarty et al., 2008). The accumulation of infected antigen presenting cells (macrophages and dendritic cells) in the regional lymph nodes leads to the development of the adaptive immune response against *M.tb*. Briefly, a type 1 T helper (T_H_1) immune response is generated and CD4 T cells secrete IFNγ, IL-2, and lymphotoxin A. Additional types of T cells also contribute to the immune response against *M.tb* infection, including CD8 T cells and γδ T cells. However, granulomas develop during experimental infection in mice deficient in these cells (D’Souza et al., 2000; Mogues et al., 2001). CD8 T cells seem to be more important, at least in the mouse model, at later stages by producing IFNγ and inducing cytotoxic activity (Lazarevic and Flynn, 2002) once the bacillary growth is stable. It has been proposed that while the early stages of infection are marked by M1 macrophage polarization, mostly due to IFNγ secretion, providing the macrophages their mycobactericidal capacity, the later stages promote a shift toward M2 polarization as a consequence of several factors, including PPARγ and STAT6 expression (Lugo-Villarino et al., 2012). This scenario would be predicted to contribute to the formation of foamy macrophages (CD36 expression) and giant cells. Although granuloma formation has long been thought to be a host-driven process, more studies indicate that there is an active role played by *M.tb* (Davis and Ramakrishnan, 2009).

Several studies have been unraveling the role of the recently identified Th17 immune response within *M.tb* granulomas. The γδ T cell population is a major source of early IL-17 during mycobacterial infections (Lockhart et al., 2006; Umemura et al., 2007), especially upon high dose infection (Hamada et al., 2008). IL-17 is most commonly associated with a pro-inflammatory response and it has been suggested to play a role during early stages of the granuloma formation promoting PMN recruitment and organization around the foci of infection (Seiler et al., 2003; Torrado and Cooper, 2010). Neutrophils and macrophages cooperate for efficient mycobacterial killing (Silva, 2010).

During the aging process there are alterations in the immune system that affect T cell functions such as decreased cytokine production (IL-2 and IFNγ), cytotoxic activity, and T cell proliferation. Animal models, more specifically the mouse model, have clearly documented the relationship between age-related decreased T cell responses and the increased risk of infection by *M.tb*, as well as the negative impact of dysregulated immune responses during *M.tb* chronic infection (Turner et al., 2001a,b; Turner and Orme, 2002). Other factors such as other diseases (e.g., diabetes mellitus), poor nutrition, and immunosuppression also impact the protective granulomatous response against *M.tb* infection during aging (Yoshikawa, 1992).

Histologically, there are different types of granulomas. Initially, epithelioid cells may be surrounded by an acellular necrotic region, with a ring of B and T cells. The granulomas can displace parenchymal tissue and may necrotize, caseate, and/or calcify. Caseous granulomas might turn calcified during chronic or latent infection. Other types of granulomas may not have a necrotic area and are composed primarily of macrophages and a few lymphocytes. Host-pathogen interactions in the granuloma over the course of infection lead to adaptive changes of the tubercle bacilli, phenotypes of the host immune cells, and levels of the immune mediators they produce. These features allow for the formation of a wide spectrum of granuloma structures even within a single human host, therefore implying the presence of several unique microenvironments for *M.tb* as well as for the immune response.

On the one hand, the granuloma’s main function is to localize and contain *M.tb* while concentrating the immune response to a limited area. On the other hand, complete eradication does not occur since *M.tb* has its own strategies to persist within the granuloma and to reactivate and escape under certain conditions. Thus *M.tb*-containing granulomas represent a unique battlefield for dictating both the host immune and bacterial response. Bacterial dissemination outside of the lungs that normally follows primary infection allows for granuloma formation in many tissue sites throughout the body, again representing different immune microenvironments. The immune microenvironment which is highly dependent on the tissue location and associated cells, growth factors, and cytokines present will determine the pattern of differentiation of the cells forming granulomas, especially the macrophages. If the antigen load at the initial site of infection and regional lymph node is large, necrosis and caseum develop which represent signatures of *M.tb* granulomas. Reactivation TB occurs in the lungs 80% of the time, whereas in 20% of cases, TB reactivates at other tissue sites (e.g., pleural space, lymph nodes, bone, kidney, etc) (Frieden et al., 2003), a consequence of the early dissemination process that occurs during primary infection. Tuberculous lymphadenitis is one of the most common forms of all extrapulmonary TB.

Differentiating the protective mechanisms involved in the mycobacterial granulomatous response from those causing tissue damage and *M.tb* spread is crucial for the TB field. The architecture, composition, function, and maintenance of granulomas are key aspects to study since they are expected to have a profound influence on *M.tb* physiology in this niche. Current knowledge about the development and maintenance of granulomas in TB is limited. However, several approaches are being pursued recently to gain more insight into the events occurring within TB granulomas. Here we review the models currently available to study the granulomatous response to *M.tb*.

## Modeling the *M.tb* Granuloma

Use of *models* to study *M.tb* granulomas that resemble those in humans is necessary because: (1) it is difficult to study human lung biopsy samples since their access is often limited; (2) human biopsy samples provide a static image that needs to be extrapolated in order to understand the dynamic processes that take place within the granuloma; and (3) *M.tb* lacks a natural host beyond humans and, therefore, surrogate models are necessary that more or less resemble human granulomas. These models are classified as *in vivo*, *in vitro*, and *in silico* models (See Table 1).

**Table 1:** **Models to study *M.tb* granulomas**.

Models	Advantages	Disadvantages
Mice*	Inexpensive, easy to handle, genetic variant strains, large number of immunological tools, and reagents available	Lack of necrosis, lack of cell structure and organization that resemble human granulomas; lack of true latency
Guinea pig/rabbit*	Easy to handle, necrosis	Limited availability of immunological tools; lack of true latency
Non-human primate*	Lesions similar to human, LTBI established	Difficult to handle, dedicated veterinarian staff required, expensive, ethical concerns
Minipig*	Pulmonary structure similar to humans, LTBI established, lesions similar to humans	Difficult to handle, dedicated veterinarian staff required, expensive, limited availability of immunological tools
Zebrafish embryo*	Easy to handle, live, real time imaging, excellent to study initial steps of granuloma formation	*M. marinum* (surrogate bacterium), lack of lung structure, lack of lymphocytes
*In vitro* human	Mimics human granuloma structure, flexible (mycobacterial strains, manipulate with, e.g., cytokines, drugs), amenable to manipulation experimentally, use for drug screening	Lack of lung structure and full tissue microenvironmental conditions
*In silico*	Inexpensive, flexible, long-term experiments with multiple, complex factors can be quickly performed; hypothesis-generating	Highly dependent on the parameters chosen, requires previous observations in different systems to extrapolate, can miss unknown factors, often not tested or proven

*In vivo models.

### *In vivo* models

Animal models are often used to study the TB granulomatous response. These models reproduce several of the processes occurring in humans, although important differences are frequently observed.

#### Mice

The mouse is the most popular animal model of *M.tb* infection. The advantages of this model include size, availability and cost, the abundant immunological tools and reagents available, and the potential for manipulation, including use of inbred, genetically modified strains (Orme, 2003). The large number of mouse models generated for infection has contributed to our understanding of the granulomatous response to *M.tb*. The most relevant is the low inoculum aerosol infection model because the aerosol route mimics the natural route of infection in humans.

Resistant strain C57BL/6 mice infected intravenously or via aerosol with moderate or low-dose inocula, respectively, develop a chronic, progressive infection with *M.tb* (North and Jung, 2004; Orme, 2005; Basaraba, 2008), with the presence of non-replicating bacilli (Rees and Hart, 1961; Wallace, 1961; Munoz-Elias et al., 2005). By 21 days post infection, a mycobacterial burden plateau is reached (∼10^5^–10^7^ CFU) followed by a steady state of infection maintained for more than 1 year (Rhoades et al., 1997). However, despite the mice surviving the infection for a long time, where the bacillary load is initially controlled due to a strong T_H_1 immune response, progressive infiltration of the lung and other tissues occurs due to changes in innate and adaptive immunity with age toward a more T_H_0–T_H_2 immune response (Cardona et al., 2000, 2003) and eventually all of the mice die from TB. This latter observation has led many to question the utility of the standard mouse model for studying granulomas in latency (Flynn, 2006).

The second mouse model widely used to study latent TB infection (LTBI) is the Cornell model. It is characterized by a high intravenous injection of 1–3 × 10^6^ virulent *M.tb* followed by isoniazid and pyrazinamide treatment for 20 weeks (McCune and Tompsett, 1956; McCune et al., 1956). Despite obtaining non-culturable *M.tb* after treatment, mice are capable of developing spontaneous or induced reactivation after 90 days (McCune et al., 1966). The lack of a standardized protocol for establishing latency with the Cornell model and the lack of stability are significant disadvantages. Factors that can alter the outcome are the genetic background of the mouse strain used, strain of *M.tb* used, inoculum preparation protocol, route of infection, and the time between infection and treatment (Scanga et al., 1999).

Given the relatively high bacterial load and progressive pathology, these two models are recognized better for resembling a chronic infection, rather than a human latent infection. However, the features of low-dose aerosol infection, healthy appearing infected mice, and episodes of reactivation are generally consistent with human LTBI. Another limitation of the standard mouse model is the lack of structure and organization of the granulomas formed which do not resemble human granulomas. Granulomas in most mouse strains are formed by loose non-necrotic cellular aggregates, a discrete fibrotic reaction, lack of encapsulation, and a strong lymphocyte presence (Rhoades et al., 1997).

Recently, new models have been developed where granulomas develop necrotic lesions in response to *M.tb* infection and thereby resemble granulomas in humans more closely (Pichugin et al., 2009; Reece et al., 2010; Driver et al., 2012; Harper et al., 2012). Intravital microscopy studies have revealed the dynamic nature of mouse TB granulomas through three-dimensional time-lapse microscopy which show activated T cells entering and moving throughout the granuloma among relatively fixed macrophages (Egen et al., 2008, 2011). A relatively new approach to study cell traffic, repopulation, and the relationship between systemic immunity and mycobacteria-containing granulomas is the granuloma transplantation model (Harding et al., 2011). In this model, a mouse liver infected with BCG or *M.tb* is transplanted by surgical insertion underneath the recipient’s kidney capsule. Interesting, new insight is being provided by this model. However, the surgical procedure, immune reaction to physical stress, length of survival of transplanted tissue, and difficulty in applying this approach to other animal species are major limitations of this model.

#### Guinea pig/rabbit

In Guinea pigs and rabbits, some granulomas are more human-like and studies in these species have yielded important insights on the development and structure of granulomas (Flynn, 2006).

Guinea pigs are highly susceptible to low-dose aerosol infection and therefore do not establish a LTBI. Rabbits, despite being relatively more resistant to *M.tb* infection, also succumb to the infection. In both models the histopathology of granulomatous lesions is similar to that seen in progressive human disease as a strong inflammatory response with fibrosis and intragranulomatous necrosis followed by mineralization or even softening and liquefaction is seen (Lenaerts et al., 2007). A down side of these models is the relative lack of immunological tools and reagents available.

#### Non-human primate

The first evidence for progression from LTBI to active disease came from the Cynomolgus macaque, a non-human primate (NHP) model (Capuano et al., 2003). NHPs, including rhesus monkeys and macaques, have been used as models for *M.tb* infection (Flynn et al., 2003). NHPs develop a disease similar to that in humans presenting a wide spectrum of human-like lesions and varied outcomes to infection. In fact, NHPs are an excellent model to study the pathogenesis and immunology of TB, as well as to screen new vaccines, diagnostic reagents, and drug treatments for pre-clinical studies. Some disadvantages of this model include cost, difficulty with handling the animals, and ethical considerations.

#### Minipig

Recently, Gil et al. have described a model of TB infection in minipigs (Gil et al., 2010). In this model, the most characteristic feature is a strong local granulomatous response that is based on the induction of a fibrotic process, where lesions are encapsulated, and intragranulomatous necrosis and calcification are present to help contain the dissemination of bacilli toward the alveolar space. The initial lesions in this model show a mixture of neutrophils, macrophages, and lymphocytes without much organization, and very few bacilli. Over time, there is an increase in fibroblast proliferation, which leads to an accumulation of myofibroblasts, and the formation of a capsule around the granuloma, which appears to be critical in preventing the spread of bacilli. The parenchyma structure of the lung, characterized by extensive interlobular and intralobular connective tissue, may play a role in the evolution of LTBI. The fact that the local pulmonary structure in minipigs is similar to that in humans makes it a viable model to study the genesis of LTBI and the granulomatous response. This model is infrequently used in the field.

#### Zebrafish embryo

Infection of zebrafish embryos with *Mycobacterium marinum*, an aquatic, close genetic relative of *M.tb*, develops organized, necrotic granulomas which appear to recapitulate human caseous granulomas. Importantly, the optical transparency of the zebrafish embryo has provided a unique tool for visualizing the dynamics of primary granuloma formation and dissemination to generate new secondary granulomas during the innate immunity phase of the infection in real time (Davis et al., 2002; Rubin, 2009). Although using *M. marinum* instead of *M.tb* is a disadvantage of the model, it provides the advantage of modeling TB in its natural host. On the other hand, the lack of lymphocytes and lung structure are limiting factors of this model.

### *In vitro* models

Several reports have described models of *in vitro* granuloma formation using peripheral blood mononuclear cells (Franklin et al., 1995; Seitzer and Gerdes, 2003; Birkness et al., 2007) involving collagen matrix gels and agarose beads or agarose-coated plates. Puissegur et al. (2004) described an *in vitro* granuloma model based on human peripheral blood mononuclear cell cultures either treated with mycobacterial antigen-coated beads or infected with mycobacteria. This model demonstrates the progressive recruitment of macrophages around live bacilli or mycobacterial antigen-coated beads, differentiation of these macrophages into multinucleated giant cells and epithelioid cells and, finally, recruitment of a ring of activated lymphocytes surrounding the granuloma structure. The epithelioid cells generated in this model have morphological and differentiation characteristics similar to those found in natural granulomas (Lay et al., 2007). This model has increased our knowledge about cell differentiation, cellular interactions and cell/bacteria interplay within the granuloma structures (Peyron et al., 2008; Russell et al., 2009). These models can be used to study the initial steps of granuloma formation and maintenance, and have the potential to address more translational aspects of human *M.tb* infection. On the other hand, they lack lung structure and thus the full tissue microenvironmental condition.

### *In silico* models

*In silico* experimentation refers to research conducted via computer simulations with models and tools that are applied to generate new hypotheses and knowledge about biological systems. As is the case for any model, the *in silico* models are systems which are applied to a specific situation, and attempt to simplify a very complex system that cannot otherwise be adequately interrogated experimentally for investigation. Computer models have been developed that describe or predict the granulomatous response outcome based on previous experimental observations and general information about TB disease (Segovia-Juarez et al., 2004; Fallahi-Sichani et al., 2011; Marino et al., 2011). These models are inexpensive, very flexible, and incorporate a number of complex parameters. They are able to ask questions that cannot be easily investigated in the laboratory and generate new hypotheses. However, they are highly dependent on the parameters chosen, require previous observations in different systems to extrapolate the results and can miss unknown factors.

## Modeling Other Granulomatous Diseases

Granulomas usually serve to protect the host from the spread of persistent microorganisms or other enduring injurious substances. Therefore, they are not restricted to *M.tb* infection; there are other bacterial, fungal, and viral infections, and even non-infectious inflammatory diseases characterized by the presence of granulomas (Sandor et al., 2003). Some of the better studied granulomatous responses relate to other infectious diseases, neoplastic processes, and autoimmune inflammatory diseases. Below is a brief summary of some of them and their main characteristics:

- Leprosy: caused by *Mycobacterium leprae* and characterized by the presence of acid-fast bacilli within macrophages in granulomas. The most commonly used models are mice, armadillos, and mathematical models (Scollard et al., 2006; Adams et al., 2012).
- Syphillis: caused by *Treponema pallidum* and characterized by the presence of gumma, a microscopic to grossly visible lesion, enclosing a wall of histiocytes, with plasma cell infiltrates and central necrosis without loss of cellular outline. The most commonly used models are hamster and mathematical models (Kajdacsy-Balla et al., 1993; Gesink Law et al., 2006).
- Sarcoidosis: caused by an unknown etiology (although mycobacterial antigens have long been implicated) and characterized by non-caseating granulomas with abundant activated macrophages. The most commonly used model is the murine model of antigen-driven granuloma formation (Samokhin et al., 2011; Yeager et al., 2012).
- Crohn’s disease: caused by immune reaction against intestinal bacteria and/or self-antigens and characterized by non-caseating granulomas in the wall of the intestine, with dense chronic inflammatory infiltrate. The most commonly used model is the murine model of chronic inflammation (Dillman et al., 2013; Tlaxca et al., 2013).
- Schistosomiasis: caused by *Schistosoma* ssp. and characterized by hepatic granuloma formation with fibrosis initiated by MHC-II-dependent, α/β^+^ CD4^+^ T lymphocytes. The most commonly used model is the SEA (*Schistosoma* egg antigen)-specific driven granuloma in mouse, monkey, and baboon animal models (Stavitsky, 2004).

Despite the extreme diversity in their etiology, several of the above diseases share general underlying histopathologic characteristics of granuloma lesions. Thus, the knowledge obtained about mycobacterial granulomas and some of the models used to study them may be useful in advancing knowledge about these other diseases.

## Conclusion

This review has described a variety of experimental models available that can help decipher the complex host-pathogen relationship that takes place within the tuberculous granuloma. Although the mycobacterial granuloma seems to be a host defense mechanism for walling off *M.tb*, the bacilli can also survive, protected from killing by immune cells, and persist in a latent form until an opportunity arises for reactivation and dissemination (Grosset, 2003). An understanding of the pathophysiology of granulomas is critical for the design of new TB drugs and vaccines. Different models are necessary to cover the wide histopathological spectrum of mycobacterial granulomas observed. Each model described in this review has and, by further development, will continue to make important contributions to TB research. The luxury of having many available models, however, must be weighed against a careful interpretation of the data obtained from them. Given the advantages and disadvantages of each model, it seems most likely that our understanding of the mycobacterial granuloma will be derived from a combination of models. Granulomas are not only present in mycobacterial infections, they can be found in many other bacterial, fungal, parasitic, or viral infections, and even in non-infectious, inflammatory granulomatous diseases (Sandor et al., 2003). Therefore, the knowledge obtained from analyzing models of mycobacterial granulomas may prove to be beneficial for uncovering mechanisms for other granulomatous diseases, including investigation of distinct infectious disease phenotypes and autoimmune as well as auto-inflammatory granulomatous diseases (e.g., Crohn’s disease or sarcoidosis).

## Conflict of Interest Statement

The authors declare that the research was conducted in the absence of any commercial or financial relationships that could be construed as a potential conflict of interest.
